# Do patients with weight loss have a worse outcome when undergoing chemotherapy for lung cancers?

**DOI:** 10.1038/sj.bjc.6601781

**Published:** 2004-04-20

**Authors:** P J Ross, S Ashley, A Norton, K Priest, J S Waters, T Eisen, I E Smith, M E R O'Brien

**Affiliations:** 1 Lung Unit, Royal Marsden Hospital, Downs Road, Sutton SM2 5PT, UK

**Keywords:** weight loss, non-small-cell lung cancer, small cell lung cancer, mesothelioma

## Abstract

To examine whether weight loss at presentation influences outcome in patients who received chemotherapy for lung cancer or mesothelioma. Multivariate analysis of prospectively collected data 1994–2001. Data were available for age, gender, performance status, histology, stage, response, toxicity, progression-free and overall survival. The outcomes of patients with or without weight loss treated with chemotherapy for small cell lung cancer (SCLC; *n*=290), stages III and IV non-small-cell lung cancer (NSCLC; *n*=418), or mesothelioma (*n*=72) were compared. Weight loss was reported by 59, 58 and 76% of patients with SCLC, NSCLC and mesothelioma, respectively. Patients with weight loss and NSCLC (*P*=0.003) or mesothelioma (*P*=0.05) more frequently failed to complete at least three cycles of chemotherapy. Anaemia as a toxicity occurred significantly more frequently in NSCLC patients with weight loss (*P*=0.0003). The incidence of other toxicities was not significantly affected by weight loss. NSCLC patients with weight loss had fewer symptomatic responses (*P*=0.001). Mesothelioma patients with weight loss had fewer symptomatic (*P*=0.03) and objective responses (*P*=0.05). Weight loss was an independent predictor of shorter overall survival for patients with SCLC (*P*=0.003, relative risk (RR)=1.5), NSCLC (*P*=0.009, RR=1.33) and mesothelioma (*P*=0.03, RR=1.92) and an independent predictor of progression-free survival in patients with SCLC (*P*=0.01, RR=1.43). In conclusion, weight loss as a symptom of lung cancer predicts for toxicity from treatment and shorter survival.

Lung cancer is the most common cancer globally with more than 1 million new cases recorded each year (Cancer Research Campaign, 2001). Approximately three-quarters of patients have non-small-cell lung cancer (NSCLC), with small cell lung cancer (SCLC) accounting for the other quarter. Survival for patients with both NSCLC and SCLC remains dismal with less than 5% of patients with NSCLC alive at 5 years and a median survival of 6–10 months for patients presenting with stage IIIB or IV disease (Cullen *et al*, 1999; Bonomi *et al*, 2000; Kelly *et al*, 2001; Smith *et al*, 2001). Similarly, the majority of patients with SCLC die within 18 months of presentation. Mesothelioma currently accounts for 10 000 deaths worldwide and this number is predicted to increase until at least 2020 (Peto *et al*, 1995). Survival is poor with a median of between 4 and 18 months in most series.

In patients with lung cancer and mesothelioma, weight loss is common at presentation and a frequent cause of patient concern. Weight loss is the result of an imbalance between energy intake and energy expenditure. Some studies have reported elevated resting energy expenditure in patients with solid cancers (Fredrix *et al*, 1991; Hyltander *et al*, 1991; Staal-van den Brekel *et al*, 1994, 1995). It was suggested that this increase is more pronounced in weight-losing patients and consequently hyper-metabolism contributed to weight loss. However, other studies have not confirmed such an increase in resting energy expenditure (Melville *et al*, 1990; Jatoi *et al*, 1999). Hyper-metabolism and weight loss have both been associated with the presence of enhanced levels of inflammatory mediators and acute phase proteins in NSCLC (Staal-van den Brekel *et al*, 1995; Simons *et al*, 1999). However, it is not known why only some tumours should result in hyper-metabolism. Many other factors may contribute to weight loss in patients with cancer, including nausea and vomiting, constipation, diarrhoea, pain, altered taste and depression, all of which may be iatrogenic or due to the cancer.

Several studies have indicated that weight loss at presentation may be an independent prognostic variable of outcome in both NSCLC and SCLC (Dewys *et al*, 1980; Stanley, 1980; Ray *et al*, 1998; Martins and Pereira, 1999; Tas *et al*, 1999), but it has not been clearly shown why this might be the case. Previous studies have not addressed whether patients with weight loss have more aggressive disease than patients without weight loss. An alternative explanation is that weight loss is associated with reduced tolerance of chemotherapy, increased toxicity and the administration of less chemotherapy overall.

The Lung Unit of the Royal Marsden Hospital (RMH) has been treating patients with NSCLC, SCLC and mesothelioma with chemotherapy over many years. This study aimed to assess whether weight loss at presentation had an influence on the toxicity patients suffered from during chemotherapy, and on whether weight loss altered the amount of chemotherapy delivered. In addition, we aimed to assess whether stabilisation of weight during treatment had any effect on outcome.

## PATIENTS AND METHODS

### Patients

This study reviewed data that had been recorded prospectively on the RMH lung unit research database between 1994 and March 2001 for patients with SCLC, stage III or IV NSCLC, or mesothelioma and treated with chemotherapy. Patients were excluded if their weight loss status at presentation was unknown or the patient did not receive a standard chemotherapy regimen within 2 months of presentation. Further exclusion criteria included prior radiotherapy and prior adjuvant or palliative chemotherapy. Patients were permitted to have radiotherapy following chemotherapy, but this variable was not included in the analysis

Patients who stated they had lost weight at the time of presentation were compared to those who denied weight loss. Parameters measured included objective and symptomatic response, treatment-related toxicity, progression free and overall survival. Within the group who had lost weight at presentation, those with continuing measured weight loss during chemotherapy were compared with those in whom weight stabilised or increased during the first 63 days of treatment. The rationale for a 63 day period for this assessment is that patients treated for NSCLC are currently treated with three cycles of chemotherapy following the findings of Smith *et al* (2001) that survival was similar for patients treated with three or six cycles of mitomycin, vinblastine and cisplatin (MVP). Patients were not given dietary advice or recommended to take dietary supplements.

### Patient assessment

Weight loss at presentation was established and recorded by direct questioning of the patient during a preliminary assessment by the doctor at their first attendance at the RMH. Patients were asked whether they had lost any weight since their illness began. Patients who reported weight loss were asked whether they knew their weight prior to the illness; by comparison with measured weight the extent of weight loss was estimated (less than or greater than 10% of preillness weight). Patients were weighed on each attendance for chemotherapy and at the outpatient clinic. Objective response to treatment was classified using the WHO/UICC response criteria following serial CT scans every 6 weeks and chest X-rays every 3 weeks (WHO, 1979). Symptoms were established by direct questioning and any change in symptoms compared to baseline was recorded on each attendance. Response of a symptom to treatment was defined as improvement in a particular symptom maintained for at least 3 weeks. Performance status was recorded at baseline and at each attendance. Toxicity was graded according to WHO toxicity criteria (WHO, 1979) by direct questioning, physical examination and measurement of full blood count, urea and electrolytes, and liver function tests.

### Treatment

Chemotherapeutic regimen depended on histology. The majority of patients treated for NSCLC were treated within the context of clinical trials with a minimum of three cycles of platinum-containing regimens, including MVP, carboplatin and vinorelbine, and docetaxel plus carboplatin (Ellis *et al*, 1995; Smith *et al*, 2001). In addition, 11 patients with NSCLC were treated with single agent vinorelbine on the basis of the results of the Elderly Lung Cancer Vinorelbine Italian Study Group (The Elderly Lung Cancer Vinorelbine Italian Study (ELVIS) Group, 1999). Patients with SCLC were treated with six cycles of established regimens (Adriamycin, cyclophosphamide and etoposide; ifosfamide, carboplatin and etoposide; carboplatin plus etoposide; MVP) (Smith *et al*, 1987, 1990; Jones *et al*, 1991; Hickish *et al*, 1998). Mesothelioma was treated with four cycles of MVP chemotherapy (Middleton *et al*, 1998).

### Statistical methods

In all analyses the three pathological types were treated independently. Response rates were compared between the patients with weight loss at presentation and those without by means of Fisher's exact test. Toxicity was graded 0–4 and a comparison between groups was carried out by means of Mann–Whitney test with trend. Comparison of the numbers of patients requiring cessation of treatment or a dose reduction because of toxicity was made by means of Fisher's exact test.

Progression free and overall survival from the date of first treatment and survival curves were generated by the method of Kaplan and Meier (1958) and compared by means of the log-rank test (Peto *et al*, 1976). The multivariate Cox's proportional hazards model (Cox, 1972) was used to calculate the relative risk (RR) of progression or death and to investigate the independent significance of prognostic variable. All *P*-values were two-sided.

## RESULTS

### Patient characteristics

This study included 780 patients treated by the RMH lung unit between 1994 and March 2001: 290 with SCLC, 418 NSCLC, and 72 with mesothelioma, with a median age of 63 years (range 27–85 years). In total, 64% of the group were male. There was no difference in the incidence of weight loss among men (62%) compared to women (57%; *P*=0.2). Patients reported weight loss more frequently with mesothelioma than with SCLC (*P*=0.01) or NSCLC (*P*=0.005) (Table 1

**Table 1:** Patient characteristics

						**Median weight (kg) of patients without weight loss**			**Median weight (kg) of patients with weight loss**	
	**Number of patients**	**Median age (range)**	**Male**	**Female**	**Number without weight loss (%)**	**Male**	**Female**	**Number with weight loss (%)**	**Male**	**Female**
SCLC	290	65 (38–85)	175	115	119 (41)	77.0	62.0	171 (59)	74.0	57.5
NSCLC	418	62 (27–82)	257	161	174 (42)	77.0	59.5	244 (58)	70.5	55.0
Mesothelioma	72	63 (42–77)	64	8	17 (24)	77.5	56.0	55 (76)	71.0	64.0

).

### Effect of weight loss at presentation on chemotherapy-related toxicity

Overall fewer patients with weight loss (315, 67%) completed three cycles of chemotherapy than those without weight loss (210, 81%; *P*<0.001). This difference was confirmed in patients with NSCLC (64% *vs* 78; *P*=0.003) (Table 2a

**Table 2:** (a) Completion of at least three cycles of chemotherapy and its relationship to weight loss, and (b) relationship between cessation of chemotherapy due to toxicity and weight loss

	**No weight loss**		**Weight loss**		
	**Number**	**Percentage**	**Number**	**Percentage**	***P***
(a)					
SCLC	100	84	131	77	0.1
NSCLC	135	78	155	64	0.003
Mesothelioma	14	72	29	53	0.05
					
(b)					
All patients	24	8	32	7	0.7
SCLC	3	3	8	5	0.5
NSCLC	18	10	18	7	0.3
Mesothelioma	3	18	6	11	0.4

) and was due to early disease progression. In contrast, in patients with SCLC there was no significant difference in the numbers of patients completing at least three cycles of chemotherapy (77 *vs* 84%; *P*=0.1) (Table 2a). Similar numbers of patients stopped treatment due to toxicity in both groups (*P*=0.7; Table 2b). In addition, overall, there were neither significant differences in frequency of dose reductions (*P*=0.6) nor treatment delays (*P*=0.2) according to weight change. However, treatment was delayed significantly more frequently in patients with weight loss associated with NSCLC than those without weight loss (9 *vs* 4%; *P*=0.04)

Patients with NSCLC with weight loss were significantly more likely to develop severe anaemia as a toxicity than those without weight loss (*P*=0.0003) (Table 3

**Table 3:** Anaemia induced by MVP chemotherapy and its relationship to weight loss

	**No weight loss**	**Weight loss**	**No weight loss**	**Weight loss**	**No weight loss**	**Weight loss**	***P***
Grade of anaemia toxicity	0	0	1–2	1–2	3–4	3–4	
SCLC	33.3%	22.6%	54.8%	62.9%	11.9%	14.5%	0.3
NSCLC	49.7%	35.8%	48.5%	57.8%	1.9%	6.5%	0.0003
Mesothelioma	31.3%	32.1%	68.8%	64.3%	0	3.6 %	0.4

). Anaemia was not more common in SCLC or mesothelioma patients with weight loss. No differences in other toxicities from chemotherapy were observed.

### Effect of weight loss at presentation on objective and symptomatic response

There was no relationship between objective response and weight loss for patients with either SCLC (*P*=0.3) or NSCLC (*P*=0.5) (Table 4

**Table 4:** Relationship between weight loss and response to chemotherapy

	**Objective response rate (%)**			**Symptom response (%)**		
	**No weight loss**	**Weight loss**	***P***	**No weight loss**	**Weight loss**	***P***
SCLC	75	69	0.3	64	52	0.06
NSCLC	37	34	0.5	60	44	0.004
Mesothelioma	29	9	0.05	65	33	0.03

). There was a lower response rate in patients with mesothelioma and weight loss (*P*=0.05). Patients with NSCLC and weight loss had significantly more symptoms at presentation than those without weight loss (*P*<0.0001) and significantly fewer symptomatic responses (44 *vs* 60%; *P*=0.004) (Table 4). In contrast, for patients with SCLC there was neither a correlation between weight loss and number of symptoms (*P*=0.3) nor a statistically significant relationship with the frequency of symptomatic response (52 *vs* 64%; *P*=0.06). For patients with mesothelioma the number of symptoms is unrelated to weight loss (*P*=0.9), but patients with weight loss reported fewer symptomatic responses (33 *vs* 65%; *P*=0.03).

### Effect of weight loss at presentation on progression-free survival

At the time of analysis disease progression was documented in 626 (80%) of the 780 patients in the study group, comprising 234 (81%) patients with SCLC, 336 (80%) patients with NSCLC and 56 (78%) patients with mesothelioma. All patients were followed up for at least 1 month and at the time of analysis 650 (83%) of the 780 patients had died. There was reduced progression-free survival in patients with weight loss and SCLC (6 *vs* 7 months; *P*=0.004) and with NSCLC (4 *vs* 6 months; *P*=0.01). However, patients with weight loss and mesothelioma did not have a statistically significant reduction in progression-free survival (3 *vs* 6 months; *P*=0.11). Progression-free survival was not statistically different in patients with weight loss greater than 10% compared to those with less than 10% weight loss with SCLC and NSCLC (data not shown). Multivariate analyses were undertaken according to tumour type, accounting for performance status, disease stage, previous surgery/radiotherapy and weight loss. Performance status and stage were the most important prognostic factors for patients with both SCLC and NSCLC (Table 5

**Table 5:** Independent factors predictive of progression-free and overall survival in patients with small cell and non-small-cell lung cancer

			**Progression free survival**		**Overall survival**	
			**RR (95% CI)**	***P***	**RR (95% CI)**	***P***
SCLC						
	Stage	Limited	1.0		1.0	
		Extensive	2.0 (1.5–2.6)	<0.001	2.4 (18–3.2)	<0.001
	PS	0	1.0		1.0	
		1	1.3 (1.1–1.6)	<0.001	1.6 (1.3–1.8)	<0.001
		2	1.8 (1.3–2.5)		2.4 (1.8–3.4)	
		3	2.4 (1.4–4.1)		3.8 (2.3–6.3)	
	Weight	No loss	1.0		1.0	
		Loss	1.4 (1.0–1.8)	0.02	1.4 (1.0–1.9)	0.003
						
NSCLC						
	PS	0	1.0		1.0	
		1	1.6 (1.3–2.0)	<0.001	1.7 (1.4–2.1)	<0.001
		2	2.7 (1.9–4.0)		3.2 (2.2–4.5)	
		3	4.5 (2.5–8.1)		5.6 (3.2–9.6)	
	Stage	III	1.0		1.0	
		IV	2.0 (1.5–2.4)	<0.001	2.0 (1.6–2.5)	<0.001
	Weight	No loss	1.0		1.0	
		Loss	1.0 (0.9–1.2)	0.4	1.3 (1.1–1.7)	0.009
						
Mesothelioma						
	PS	0			1.0	
		1			1.6 (1.2–2.3)	0.004
		2			2.7 (1.4–5.1)	
		3			4.3 (1.6–11.6)	
	Weight	No loss			1.0	
		Loss			1.9 (1.1–3.5)	0.03

). Weight loss resulted in an increased RR of progression (1.4, 95% confidence intervals 1.0–1.8) for patients with SCLC. In contrast, weight loss was not a prognostic factor of progression-free survival for patients with NSCLC. No factors were significant predictors of progression-free survival in patients with mesothelioma.

### Effect of weight loss at presentation on survival

**Figure 1:**
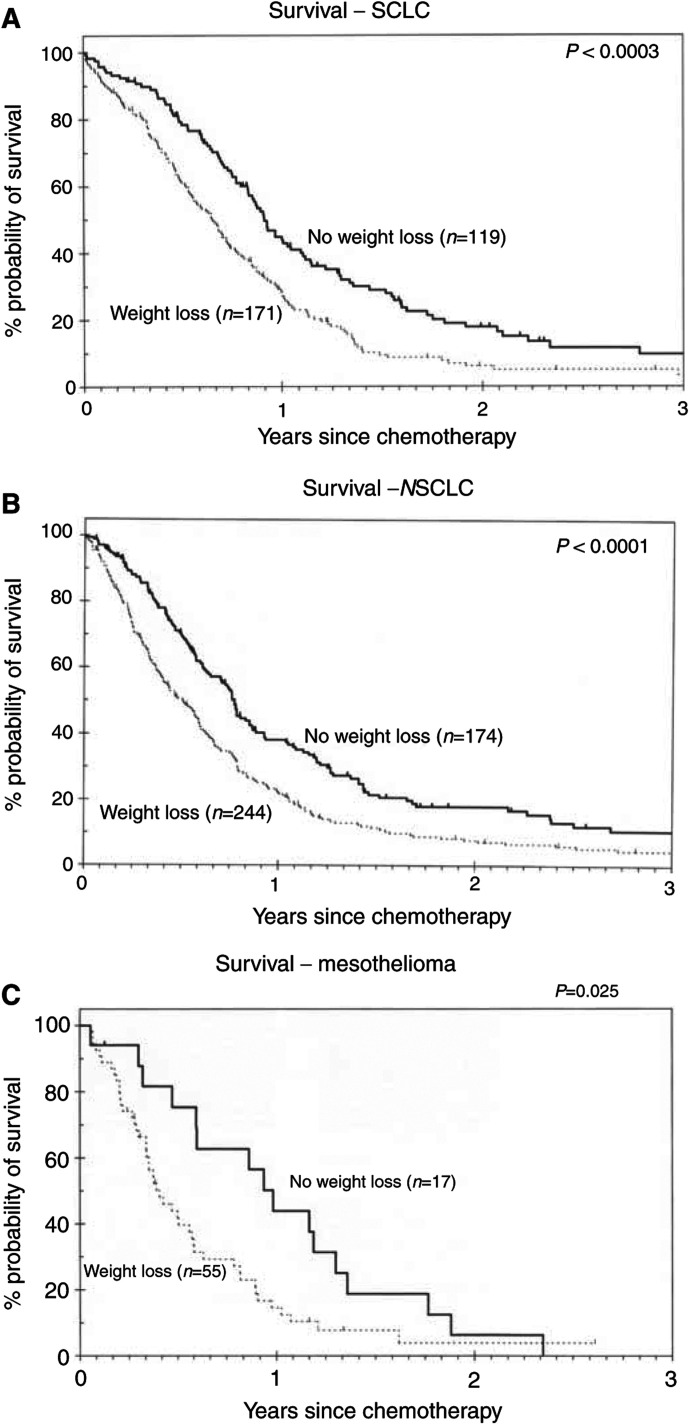
Survival in patients with compared to those without weight loss associated with small cell lung cancer (**A**), non-small-cell lung cancer (**B**) and mesothelioma (**C**).

Overall survival was significantly shorter for patients with weight loss compared to those without weight loss with SCLC (8 *vs* 11 months; *P*=0.0003), NSCLC (6 *vs* 9 months; *P*<0.0001) and mesothelioma (5 *vs* 12 months; *P*=0.025) (Figure 1A–C

). Multivariate analysis demonstrated that performance status was prognostic for patients with SCLC (*P*<0.001), NSCLC (*P*<0.001) and mesothelioma (*P*=0.004) (Table 5). In addition, stage was prognostic for patients with SCLC and NSCLC. Weight loss remained a prognostic factor for SCLC (RR 1.5, 95% CI 1.0–1.9), NSCLC (RR 1.3, 95% CI 1.1–1.7) and mesothelioma (RR 1.9, 95% CI 1.1–3.5). Female gender predicts for improved overall survival (RR 0.8, 95% CI 0.6–1.0, *P*=0.03) and progression-free survival (RR 0.8, 95% CI 0.6–1.0, *P*=0.04) in patients with NSCLC.

### Effect of stabilisation of presentation weight loss on progression-free and overall survival

Data were not available on whether patients with weight loss had received nutritional intervention of any kind. However, as patients were weighed prior to each cycle of chemotherapy data on weight stabilisation were available. Therefore, we examined whether weight stabilisation during the first 63 days after presentation in patients who had lost weight at presentation improved outcome.

**Figure 2:**
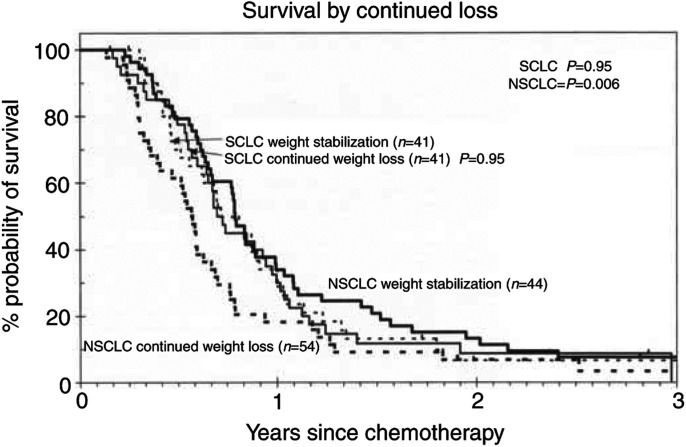
Survival in patients in whom weight stabilised during treatment with chemotherapy compared to patients who continued to lose weight.

Weight was recorded over the first 63 days in 198 of the 470 patients presenting with weight loss. For patients with SCLC weight stabilisation did not significantly improve progression-free (*P*=0.8) or overall survival (*P*=0.95) in comparison to those who continued losing weight (Figure 2

). In contrast, weight stabilisation for patients with NSCLC resulted in a significant improvement in both progression-free and overall survival. Progression-free survival increased from 5 to 7 months (*P*=0.01) and overall survival from 7 to 9 months (*P*=0.006) (Figure 2). Only three patients with mesothelioma had weight stabilisation during treatment and this did not affect survival.

## DISCUSSION

An important issue for physicians treating patients with lung cancer and mesothelioma is optimising symptomatic care in view of the modest survival benefits of cytotoxic chemotherapy. Weight loss is recognised to occur frequently and has been identified as a prognostic factor in NSCLC (Dewys *et al*, 1980; Stanley, 1980; Hoeltgen *et al*, 1983), SCLC (Dewys *et al*, 1980; Wolf *et al*, 1991; Bremnes *et al*, 2003) and mesothelioma (Herndon *et al*, 1998; Edwards *et al*, 2000). In contrast, other studies have not confirmed weight loss as prognostic for either NSCLC (O’Connell *et al*, 1986; Sorensen *et al*, 1989; Paesmans *et al*, 1995), SCLC (Paesmans *et al*, 2000; Christodolou *et al*, 2002), or mesothelioma (Curran *et al*, 1998). This study has demonstrated that weight loss at presentation is an independent prognostic factor for survival of patients’ with NSCLC, SCLC and mesothelioma.

This is the first study to examine the relationship between weight loss, toxicity, delivery of chemotherapy, response to treatment and prognosis in patients with lung cancer and mesothelioma. In NSCLC weight loss is associated with the delivery of fewer cycles of chemotherapy and more treatment delays, together with an increased incidence of anaemia as a toxicity. In addition, weight loss was associated with fewer symptomatic responses, although there was no significant difference in the rate of objective response. Furthermore, patients whose weight stabilised on treatment had significantly better progression-free and overall survival than those with continued weight loss. Similarly, in patients with mesothelioma weight loss was associated with fewer patients completing at least three cycles of chemotherapy and significantly lower rates of both symptomatic and objective responses. In contrast, weight loss associated with SCLC neither affected the number of patients completing at least three cycles of chemotherapy, the incidence of toxicity nor the response rate. Moreover, weight stabilisation on treatment neither improved progression-free nor overall survival. Thus, different patterns of outcomes were identified for patients with weight loss associated with NSCLC (and mesothelioma) compared to those with SCLC.

These differences may be due to differences in the mechanism of weight loss between the two diseases. Support for this hypothesis is suggested by a study indicating that weight loss in SCLC is associated with a greater increase in resting energy expenditure adjusted for fat-free mass than NSCLC associated weight loss (Staal-van den Brekel *et al*, 1997). This suggests changes in carbohydrate compared with fat metabolism specifically related to the biology of SCLC. Furthermore, it is possible that such changes in metabolism may have an adverse effect on outcomes for patients with SCLC irrespective of treatment.

Nonetheless, it remains to be demonstrated whether weight loss is simply a marker of patients with a poor prognosis or whether it independently reduces the ability of some patients to be treated effectively with chemotherapy. The response to chemotherapy may be altered by weight loss; even a 5% weight loss alters measurable physiological parameters, such as immune response, lung and cardiac function tests and autonomic regulation (Jones, 1992). In the only large group of patients treated with chemotherapy where the detailed significance of weight loss is known, it has been demonstrated that weight loss of only 5% at presentation had a significant adverse effect on survival (Deng *et al*, 1995). If the latter were the case it might be predicted that conventional means of nutritional intervention could be an important adjunct to treatment. A number of studies have evaluated enteral (Evans *et al*, 1987; Ovesen *et al*., 1993) and parenteral (Klein *et al*., 1986; McGeer *et al*., 1990) nutritional support in patients receiving treatment for cancer and suggested no benefit from such interventions. However, these studies were underpowered to adequately assess the effect. Therefore, a well-designed study to evaluate the benefit of nutritional support in patients with weight loss receiving chemotherapy is needed. Given that weight stabilisation was associated with improved survival in NSCLC, such a study should be conducted in patients with NSCLC (and mesothelioma).

The only toxicity that occurred significantly more frequently in patients with weight loss was anaemia. Intriguingly, we have previously observed that a nadir haemoglobin level less than 12 g dl^−1^ during chemotherapy treatment for SCLC and NSCLC was associated with poorer survival (Waters *et al*., 2002). Thus, the increased incidence of chemotherapy-related anaemia in patients with weight loss may contribute to the inferior survival.

Weight loss is an important issue with multivariate analysis demonstrating an increased risk of death in patients with SCLC, NSCLC and mesothelioma. In addition, patients with NSCLC had more symptoms at presentation and in all patients there was a trend towards reduced symptomatic benefit from chemotherapy. However, at least for patients with NSCLC where weight was stabilised, the data suggest a better outcome can be hoped for. This study emphasises the requirement for randomised studies of nutritional intervention initially in patients with NSCLC.
